# Unusual hematoma after spinal anesthesia

**DOI:** 10.11604/pamj.2018.31.203.15977

**Published:** 2018-11-22

**Authors:** Jaouad Laoutid, Omar Laghzaoui

**Affiliations:** 1 Department of Anesthesia Critical Care, Military Hospital Moulay Ismail, Meknes, Morocco; 2 Service of Gynecology and Obstetrics, Military Hospital Moulay Ismail, Meknes, Morocco

**Keywords:** Spinal anesthesia, hematoma, obesity

## Image in medicine

A 45-year-old female, obese with a BMI of 39.5 was scheduled to undergo hysteroscopy for endometrial hyperplasia under spinal anesthesia. She was diagnosed with well controlled diabetes and hypertension. She took neither antiaggregant nor anticoagulants and biological values were normal. In the operating theater, in sitting position, we could not detect the spinous process so we used the line of iliac crests as landmark for the level L4-L5. After several unsuccessful attempts to perform the spinal anesthesia at different levels, a swelling rapidly progressive and extensive appeared at the points of puncture. The intervention was reported and immediate lumbar scan showed a big subcutaneous hematoma in front of the last thoracic vertebras measuring 12x28x20 cm. Hemodynamic values, hemoglobin and the size of the hematoma remained stable and surgical abstention was decided. Hematoma was caused by probable injury of the posterior branch of spinal artery, favored by difficult spinal anesthesia for patient obesity and lumbar hyperlordosis, hence the interest of ultrasound localization both for level L4-L5 and for spinous process in obese patient. Three weeks later, hematoma was completely resolved and hysteroscopy performed under general anesthesia. Biopsy revealed endometrial adenocarcinoma.

**Figure 1:**
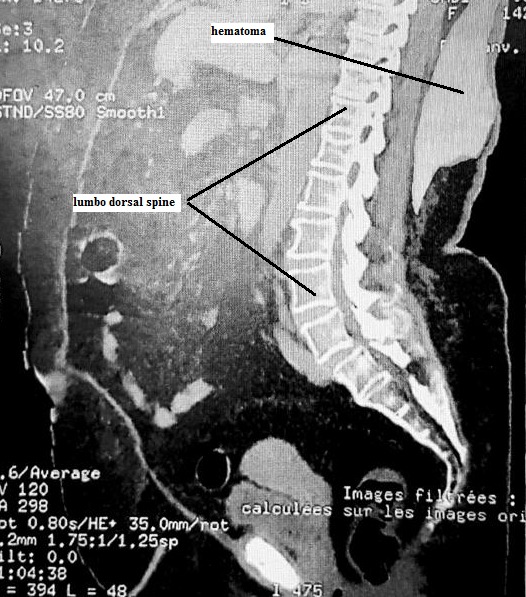
Lumbo dorsal spine and hematoma

